# HIV infection is not associated with perioperative blood loss in patients undergoing total hip arthroplasty

**DOI:** 10.1186/s13018-022-03055-y

**Published:** 2022-03-09

**Authors:** Qifeng Wei, Gaorui Cai, Guoneng Chen, Maoye Shen, Ya Gao, Xianjia Ning, Jinghua Wang, Wenxue Jiang

**Affiliations:** 1 Department of Orthopedics, The Third People’s Hospital of Shenzhen, 29 Bulan Road, Shenzhen, 518112 Guangdong Province China; 2 Center of Clinical Epidemiology, The Third People’s Hospital of Shenzhen, 29 Bulan Road, Shenzhen, 518112 Guangdong Province China; 3 Laboratory of Epidemiology, Tianjin Neurological Institute, 154 Anshan Road, Heping District, Tianjin, 300052 China

**Keywords:** Perioperative blood loss, Total hip arthroplasty, Osteonecrosis of the femoral head, Hidden blood loss, Dominant blood loss

## Abstract

**Background:**

Patients with HIV have a higher prevalence of thrombocytopenia than those without HIV infection, increasing their risk of substantial perioperative blood loss (PBL) during total hip arthroplasty (THA). This study aimed to evaluate PBL risk factors in HIV-infected patients undergoing THA.

**Methods:**

Eighteen HIV+ patients (21 hip joints) and 33 HIV− patients (36 joints) undergoing THA were enrolled in this study. PBL was calculated using the Gross equation, which comprises total blood loss (TBL), dominant blood loss (DBL), and hidden blood loss (HBL). Risk factors for post-THA PBL in both patient populations was evaluated using multivariable linear regression.

**Results:**

At baseline, the HIV+ patients were younger, more likely to be male and to have elevated hemoglobin and albumin levels, and lower erythrocyte sedimentation rates than HIV− patients. There were no differences in the T-lymphocyte subsets or coagulation function between the two groups. Age and albumin level were identified as potential HBL risk factors after THA, and albumin level was associated with higher TBL. The unadjusted linear regression analysis showed that the HBL and TBL were significantly higher in HIV+ patients than in HIV− patients. However, after adjusting for other factors, no differences in DBL, HBL, or TBL were observed between HIV− and HIV+ patients.

**Conclusion:**

PBL was similar in both groups undergoing THA, regardless of their HIV-infection status. THA surgery is a safe and effective procedure in HIV+ patients.

## Introduction

With the widespread use of highly active antiretroviral therapy (HAART), HIV has been transformed from a devastating disease to a chronic condition [1, 2]. Moreover, the incidence of HIV-related deaths and HIV-associated opportunistic infections has notably decreased. As a result, previously unrecognized complications of long-term HIV infections are having an increased impact on the patient’s quality of life [3, 4].

Osteonecrosis of the femoral head (ONFH) has been recognized as an important complication of long-term HIV infection [5–9]. The estimated incidence of ONFH among patients with HIV ranges from 0.3 to 3.7 cases per 1000 person-years, which is much higher than the estimated incidence among the general population [10, 11]. Moreover, asymptomatic ONFH is also common among patients with HIV. Previous studies reported that 4.4% of asymptomatic patients with HIV exhibited signs of ONFH on MRI, while the rate was 1.7% among those without HIV [10, 12].

Total hip arthroplasty (THA) is an effective surgical treatment for ONFH, with perioperative blood loss (PBL) being a key factor in postoperative recovery. However, in HIV+ patients, the virus invades the platelets, leading to decreased platelet counts and secondary coagulation dysfunction; the overall prevalence of thrombocytopenia in this population is 4.5–26.2% [13]. The peripheral destruction of platelets and decreased platelet production are two important mechanisms of thrombocytopenia in HIV+ patients [14, 15]. Although PBL is a major concern in THA for HIV+ patients, published studies have not estimated PBL in these patients. Hence, this study investigated PBL risk factors among HIV-infected patients undergoing THA.

## Methods

### Study design

From August 2020 to April 2021, we continuously enrolled patients with or without who underwent primary THA surgery at the Shenzhen Third People's Hospital. The inclusion criteria for HIV+ patients were as follows: (1) diagnosis of HIV by laboratory examination, including HIV 1/2 antibody screening, flow cytometry for T lymphocyte, and HIV-RNA nucleic acid virus load, in combination with a personal history; (2) history of receiving HAART for more than 6 months before the procedure; (3) history of femoral neck fracture and femoral head necrosis caused by osteoarthritis, drug, alcohol, etc.; and (4) age ≥ 18 years. We included HIV− patients that were (1) aged ≥ 18 years, and had (2) femoral neck fracture and femoral head necrosis caused by osteoarthritis, drug, alcohol, etc. We excluded patients with (1) a previous history of hip surgery at the same site; (2) complication with infection or tumor at the surgical site of the hip joint; and (3) a history of abnormal nutritional, immune, and coagulation functions. All the patients had a history of prior ipsilateral hip surgery; had a hip joint to be operated on that was also complicated with an infection or tumor; or had abnormal nutritional, immune, or coagulation functions were excluded.

### Perioperative process

HIV patients had received standardized HAART before the procedure. Ten patients received TDF + 3TC + EFV treatment for more than 2 years. Two patients were treated with TDF + 3TC + LPV / r for 2–3 years. Two patients were treated with TDF + 3TC + RAL for about 1 year, and four patients were treated with ABC + 3TC + RAL for 1–2 years.

All operations were performed by a senior surgeon. After general anesthesia and endotracheal intubation, the posterolateral approach was used to perform THA. The same acetabular cup and femoral component (Betacup®; Link, Germany) were used in both groups; 5% glucose injection plus tranexamic acid 0.5 g was used 5–10 min before and during the procedure in both groups. All patients were injected subcutaneously with 4100 IU of low-molecular-weight heparin calcium 12 h before the procedure, and once a day after 12 h postoperatively. Intravenous transfusion of leukocyte-reduced red blood cells, virus-inactivated plasma, or apheresis platelet was used as appropriate for patients who needed blood transfusion during the operation.

Postoperatively, negative-pressure drainage tubes were placed at the surgical site. The tubes were removed 24–48 h after surgery.

### PBL evaluation

Data were collected regarding intraoperative blood loss, blood transfusions, and 24-h postoperative wound drainage. PBL comprised estimated total blood loss (TBL), which was calculated according to the serum hematocrit (HCT) using the Gross equation [16], in addition to dominant blood loss (DBL) and hidden blood loss (HBL). The equations are shown as follows:

Estimated TBL = preoperative blood volume × (preoperative HCT − postoperative HCT)/mean HCT, where blood volume (L) = *K*1 × height (m)^3^ + *K*2 × weight (kg) + *K*3. For males, *K*1 = 0.3669, *K*2 = 0.03219, and *K*3 = 0.6041; for females, *K*1 = 0.3561, *K*2 = 0.03308, and *K*3 = 0.1833. Preoperative HCT was determined 1 day before the procedure at the Shenzhen Third People's Hospital, and postoperative HCT was determined 5 days postoperatively because volume distribution reached a steady state.

$$\begin{aligned} & {\text{TBL }} = {\text{ estimated}}\,{\text{TBL }} + {\text{ blood}}\,{\text{transfusion}}\,{\text{volume}} \\ & {\text{DBL }} = {\text{ intraoperative}}\,{\text{blood}}\,{\text{loss }} + {\text{ postoperative}}\,{\text{blood}}\,{\text{ loss}} \\ & {\text{HBL }} = {\text{ TBL}} - {\text{DBL}} \\ \end{aligned}$$

### Data collection

Baseline data (age, sex, height, weight, and body mass index) were routinely collected preoperatively. T-lymphocyte subsets (absolute counts of CD3^+^ T cells, CD4^+^ T cells, and CD8^+^ T cells) were also assessed preoperatively. Other clinical information, including levels of hemoglobin, albumin, and high-sensitivity C-reactive protein, HCT, erythrocyte sedimentation rate (ESR), and indicators of coagulation function (prothrombin time, international normalized ratio, and fibrinogen concentration) were measured before and after the operation. Nutrition status was assessed using the European Society for Clinical Nutrition and Metabolism guidelines [17].

### Statistical analysis

Continuous data are presented as means and standard deviations or as medians and interquartile ranges. Student’s *t*-test was used to analyze between-group differences if the distribution was normal as assessed by the Shapiro–Wilk test. The Mann–Whitney U test was applied for non-normal continuous data. Categorical data are presented as frequencies and percentages; and the Fisher’s exact test was used to compare between-group differences. Univariable analysis was conducted to assess the potential risk factors associated with PBL. Multivariable linear regression analysis was used to evaluate blood loss differences between patients with and without HIV infection; independent variables were chosen from the univariable analysis (those with *P* values < 0.05). All analyses were conducted using SPSS for Windows (version 22.0; SPSS, Chicago, IL, USA). *P* < 0.05 was considered statistically significant.

## Results

### Patient baseline characteristics

**Fig. 1:**
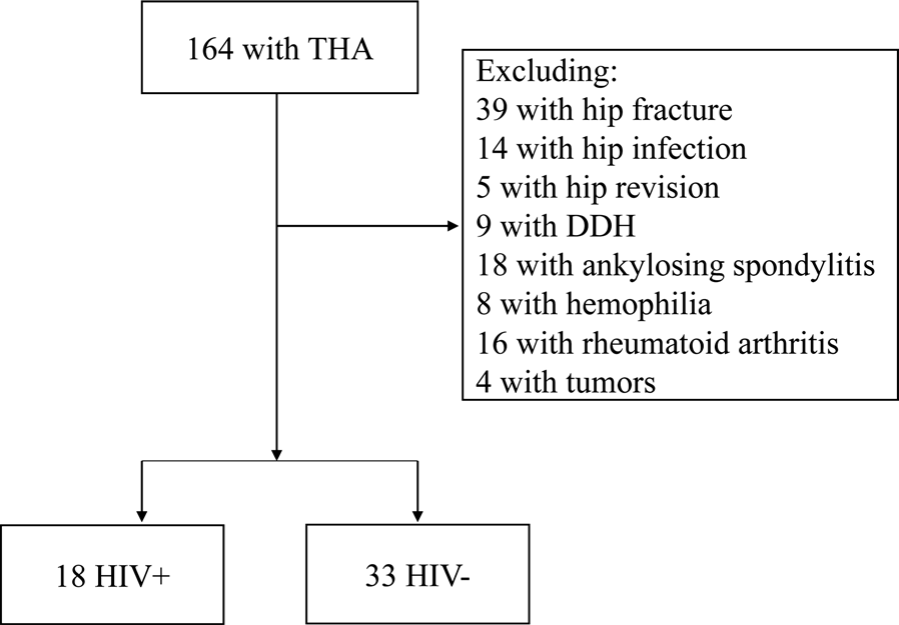
Flow chart of patients selection

A total of 51 patients were enrolled in the study, including 18 (21hip) who were HIV+ and 33 (36 hip) who were HIV− (Fig. 1). Table 1 shows the baseline characteristics and preoperative clinical information for the HIV− and HIV+ patients. Among the HIV− patients, 55.6% were female, whereas female patients accounted for only 9.5% of HIV+ patients. The average ages of the two groups were 60.97 (HIV−) and 43.19 (HIV+) years. The HIV+ patients were more likely to have higher hemoglobin and albumin levels, and lower ESR and drainage volume than the HIV− patients. There were no differences in blood transfusion volume, T-lymphocyte subsets, and coagulation function indicators between the two groups.

**Table 1:** Baseline characteristic of patients with total hip arthroplasty

Factors	HIV−	HIV+	*P* value
Gender, *n* (%)			0.001
Men	16 (44.4)	19 (90.5)	
Women	20 (55.6)	2 (9.5)	
Hypertension,* n* (%)			0.832
Yes	13 (36.1)	7 (33.3)	
No	23 (63.9)	14 (66.7)	
Diabetes, *n* (%)			0.070
Yes	6 (16.7)	8 (38.1)	
No	30 (83.3)	13 (61.9)	
BMI group			0.484
Low-weight	5 (13.9)	4 (19.0)	
Normal	23 (63.9)	10 (47.6)	
Over-weight	8 (22.2)	7 (33.3)	
Age, mean ± SD, years	60.97 ± 17.37	43.19 ± 12.06	< 0.001
Hb, mean ± SD, g/L	125.25 ± 22.87	137.67 ± 16.9	0.023
HCT, mean ± SD, g/L	38.92 ± 10.37	40.19 ± 4.82	0.600
ALB, mean ± SD, g/L	39.46 ± 3.96	42.63 ± 3.54	0.004
ESR, median (IQR), mm/h	40.0 (52)	18.5 (20.5)	0.088
hs-CRP, median (IQR), mg/L	5.6 (27.0)	4.7 (7.3)	0.330
BMI, mean ± SD, kg/m^2^	22.66 ± 4.93	22.35 ± 3.81	0.687
Drainage volume, Median (IQR), ml	140 (180)	100 (0)	0.029
Blood transfusion volume, Median (IQR), ml	580 (290)	580 (0)	0.451
Nutrition status, Median (IQR)	1 (2)	1 (0)	0.733
PT, mean ± SD, s	13.13 ± 1.13	12.81 ± 0.69	0.495
INR, mean ± SD	0.99 ± 0.11	0.96 ± 0.07	0.305
Fibrinogen, mean ± SD, g/L	3.81 ± 1.11	4.00 ± 1.05	0.540
Platelet count, mean ± SD, 10^9^/L	244.13 ± 84.68	258.52 ± 58.69	0.717

Hb: hemoglobin; HCT: hematocrit; ALB: albumin; ESR: erythrocyte sedimentation rate; hs-CRP: high sensitivity C-reactive protein; BMI: body mass index; PT: prothrombin time; INR: International normalized ratio

### Univariable analysis of post-THA blood loss risk factors

Table 2 shows the single factor analysis of potential PBL risk factors for all patients. HIV infection was significant associated with blood loss, including DBL, HBL, and TBL (all *P* < 0.05). Compared with HIV− group, the levels of DBL, HBL and TBL were higher in HIV+ group. Moreover, diabetes, and BMI group were found to be associated with DBL. Age, HB, ALB, and hs-CRP level were potential risk factors of HBL. ALB and hs-CRP level were associated with TBL.

**Table 2:** Univariable analysis of the risk factors of blood loss in total hip arthroplasty

Risk factors	HBL	*P*	DBL	*P*	TBL	*P*
	Median (IQR)/*β* (95% CI)		Median (IQR)/*β* (95% CI)		Median (IQR)/*β* (95% CI)	
HIV status		0.010		0.016		0.014
HIV−	1668 (1000)		400 (300)		2107 (1177)	
HIV+	2150 (1065)		650 (550)		2626 (1239)	
Gender		0.054		0.494		0.131
Men	2083 (1012)		400 (488)		2546 (1157)	
Women	1666 (1073)		457 (388)		2110 (1142)	
Hypertension		0.798		0.158		1.000
Yes	1859 (1113)		500 (650)		2230 (1088)	
No	1889 (1077)		400 (393)		2194 (1183)	
Diabetes		0.570		0.001		0.427
Yes	2016 (1093)		575 (490)		2663 (1207)	
No	1727 (1017)		400 (388)		2152 (1106)	
BMI group		0.523		0.040		0.327
Low-weight	2036 (706)		465 (905)		2501 (1455)	
Normal	1708 (1019)		400 (300)		2107 (1152)	
Over-weight	2200 (1473)		700 (650)		2663 (1149)	
Age	− 16.7 (− 30.9, − 2.5)	0.022	1.2 (− 8.35, 10.8)	0.802	− 14.0 (− 32.6, 4.5)	0.136
Hb	12.6 (0.93, 24.3)	0.035	− 6.2 (− 13.9, 1.5)	0.112	7.8 (− 7.5, 23.1)	0.313
ALB	109.8 (51.7, 167.9)	< 0.001	24.8 (− 16.8, 66.5)	0.238	123.6 (47.5. 199.6)	0.002
ESR	− 5.9 (− 17.3, 5.4)	0.296	− 4.6 (− 12.1, 3.0)	0.229	− 8.4 (− 22.9, 6.1)	0.253
hs-CRP	− 10.8 (− 20.2, − 1.4)	0.025	− 2.4 (− 8.7, 4.0)	0.460	− 12.3, (− 24.4, − 0.2)	0.046
Nutrition status	− 10.4 (− 220.5, 199.7)	0.921	39.6 (− 86.1, 165.2)	0.530	39.2 (− 227.5, 305.9)	0.769
PT	483.6 (− 240, 1207.0)	0.162	53.5 (− 340.6, 447.6)	0.762	573.1 (− 222.8, 1296.9)	0.142
INR	5135.0 (− 2938.2, 13,208.7)	0.181	318.3 (− 4050.6, 4687.1)	0.871	5463.5 (− 3145.2, 14,052.2)	0.182
Fibrinogen	− 369.0 (− 1001.8, 263.8)	0.216	− 25.8 (− 362.4, 310.7)	0.864	− 394.8 (− 1067.6, 277.9)	0.213
Platelet count	− 0.4 (− 3.9, 3.0)	0.814	− 1.1 (− 3.3, 1.1)	0.332	− 0.9 (− 5.3, 3.4)	0.663

Hb: hemoglobin; ALB: albumin; ESR: erythrocyte sedimentation rate; hs-CRP: high sensitivity C-reactive protein; BMI: body mass index; PT: prothrombin time; INR: International normalized ratio

### Linear regression of blood loss in HIV+ and HIV− patients

Table 3 shows the linear regression analysis of blood loss in both patient groups. The unadjusted linear regression showed that HBL and TBL were significantly higher in HIV+ patients than in HIV− patients. However, when adjusted for variables with *P* value < 0.05 in the univariable analysis, no between-group differences in DBL, HBL, or TBL were observed.

**Table 3:** The liner regression of blood loss between HIV− and HIV+

Blood loss	Unadjusted		Adjusted	
	*β* (95% CI)	*P*	*β* (95% CI)	*P*
DBL	362.9 (28.1, 697.7)	0.034	289.7 (− 46.3, 625.7)	0.090
HBL	726.7 (222.0, 1231.4)	0.006	580.4 (− 24.4, 1185.2)	0.060
TBL	855.3 (207.2, 1503.5)	0.011	662.6 (− 56.9, 1382.1)	0.070

DBL: Dominant blood loss; HBL: hidden blood loss; TBL: total blood loss

## Discussion

In this study, we enrolled 18 patients (21 joints) who were HIV+ and 33 (36 joints) who were HIV−. Multivariable linear regression failed to demonstrate any significant difference in PBL between the two groups.

With the increasing maturity of artificial joint technology, THA has become a safe and effective surgical treatment for end-stage hip disease [18]. In recent years, HIV+ patients have received HAART, and awareness regarding the complications of HAART is also increasing, including osteoporosis and avascular necrosis of femoral head [19, 20]. Currently, THA surgery is a routine surgical treatment for HIV-infected patients with end-stage hip disease. In patients undergoing THA, excessive PBL may lead to increased incidences of postoperative fever, anemia, hypoproteinemia, wound and joint infections, lower extremity deep venous thrombosis, pulmonary embolisms, and other complications. These complications affect the patient’s short-term recovery and long-term joint function. Thus, orthopedists need to provide correct, active, and effective control of PBL.

Platelets have been reported to interact with the HIV-1 virus, viral membrane proteins, or dysregulated circulating inflammatory molecules resulting from HIV-1 infection [21]. Thus, long-term HIV infections often lead to thrombocytopenia and idiopathic thrombocytopenic purpura (ITP). A study conducted in Brazil revealed that 63.6% of HIV patients had ITP, and 25.5% had platelet production deficiencies that were secondary to HIV infection [22]. An earlier study reported that the cross-reactivity between viral envelope glycoprotein 120 and platelet glycoprotein IIIa promotes platelet capture and lysis in the reticuloendothelial system of the spleen or early apoptosis, both of which result in ITP [14]. Moreover, during the advanced stages of HIV infection, the HIV-1 virus impairs the signaling of colony-forming units associated with megakaryocyte growth and further disrupts megakaryocytic maturation [23]. However, with the widespread use of HAART, the loading of the HIV-1 virus can be stably suppressed, transforming HIV infection from a disease of high mortality into a chronic disease. Many studies have found that the prevalence of thrombocytopenia decreases with higher CD4^+^ T-lymphocyte counts; the prevalence drops to 0 in patients with CD4^+^ T lymphocyte counts of > 350 cells/μL [24, 25]. In our study, thrombocytopenia and its corresponding complications were not observed. Thus, the difference in PBL between HIV− and HIV+ patients was insignificant.

Several management measures are used to prevent PBL, which may be operative, hemostatic, or blood-related. During the intraoperative period, surgeons need to ensure accurate anatomical positioning, timely hemostasis, and minimal peeling of the surrounding tissues. Reducing soft tissue and bone damage does not only reduce intraoperative DBL, it also accelerates the postoperative recovery of muscle function. Soft tissue injury can be minimized by avoiding repeated pulling of the muscles and other soft tissues [18]. During the postoperative period, early active rehabilitation can accelerate the recovery of muscles and other soft tissues. Through appropriate active exercise, combined with passive exercise, significant reductions in HBL and improved patient prognoses can be achieved [26]. Tranexamic acid is an effective hemostatic agent for reducing intraoperative bleeding. This agent can be locally applied within 10 min of creating an incision or prior to incision closure to achieve less HBL, without any adverse effects during the perioperative period [27, 28]. Blood management is another important measure for reducing PBL. Orthopedic surgeons should evaluate the preoperative hemoglobin status of patients to avoid anemia, which leads to a deterioration of the body postoperatively and increases infection and mortality rates; they should also apply measures to decrease the postoperative recovery period and shorten the hospital stay [29].

The present study has several limitations. First, our study involved a small number of patients; future studies need larger sample sizes to evaluate the validity of the present results. Second, the underlying mechanisms of increased PBL in HIV+ patients are unknown. Thus, exploring the reasons for higher PBL in HIV+ patients is necessary. Third, this study was a single-center investigation, which could reduce the generalizability of the results. Lastly, sex imbalance was inevitable because the HIV+ patients in this study were mostly male homosexuals, while the proportion of men in HIV− group was notably less.

## Conclusion

In conclusion, there were no differences in post-THA blood loss between patients with and without HIV infection. This study also confirmed that THA is a safe and effective modality in HIV+ patients; the procedure is not associated with an increased risk of bleeding in HIV+ patients and has the same level of surgical effectiveness in HIV+ and HIV− patients.

## Data Availability

The datasets used and/or analysed during the current study are available from the corresponding author on reasonable request.

## Abbreviations

ONFH: Osteonecrosis of the femoral head
HIV: Human immunodeficiency virus
PBL: Perioperative blood loss
THA: Total hip arthroplasty
TBL: Total blood loss
HBL: Hidden blood loss
HAART: Highly active antiretroviral therapy
HCT: Hematocrit
BV: Blood volume
DBL: Dominant blood loss
Hb: Hemoglobin
ALB: Albumin
ESR: Erythrocyte sedimentation rate
SDs: Standard deviations
IQRs: Interquartile ranges
ITP: Idiopathic thrombocytopenic purpura
